# Conserved Substitution Patterns around Nucleosome Footprints in Eukaryotes and Archaea Derive from Frequent Nucleosome Repositioning through Evolution

**DOI:** 10.1371/journal.pcbi.1003373

**Published:** 2013-11-21

**Authors:** Tobias Warnecke, Erin A. Becker, Marc T. Facciotti, Corey Nislow, Ben Lehner

**Affiliations:** 1 Bioinformatics and Genomics Program, Centre for Genomic Regulation (CRG) and UPF, Barcelona, Spain; 2 Universitat Pompeu Fabra (UPF), Barcelona, Spain; 3 Microbiology Graduate Group, University of California, Davis, Davis, California, United States of America; 4 Department of Biomedical Engineering, University of California, Davis, Davis, California, United States of America; 5 Genome Center, University of California, Davis, Davis, California, United States of America; 6 Department of Pharmaceutical Sciences, University of British Columbia, Vancouver, British Columbia, Canada; 7 EMBL-CRG Systems Biology Unit, Centre for Genomic Regulation (CRG), Barcelona, Spain; 8 Institució Catalana de Recerca i Estudis Avançats, Centre for Genomic Regulation (CRG) and UPF, Barcelona, Spain; Weizmann Institute of Science, Israel

## Abstract

Nucleosomes, the basic repeat units of eukaryotic chromatin, have been suggested to influence the evolution of eukaryotic genomes, both by altering the propensity of DNA to mutate and by selection acting to maintain or exclude nucleosomes in particular locations. Contrary to the popular idea that nucleosomes are unique to eukaryotes, histone proteins have also been discovered in some archaeal genomes. Archaeal nucleosomes, however, are quite unlike their eukaryotic counterparts in many respects, including their assembly into tetramers (rather than octamers) from histone proteins that lack N- and C-terminal tails. Here, we show that despite these fundamental differences the association between nucleosome footprints and sequence evolution is strikingly conserved between humans and the model archaeon *Haloferax volcanii*. In light of this finding we examine whether selection or mutation can explain concordant substitution patterns in the two kingdoms. Unexpectedly, we find that neither the mutation nor the selection model are sufficient to explain the observed association between nucleosomes and sequence divergence. Instead, we demonstrate that nucleosome-associated substitution patterns are more consistent with a third model where sequence divergence results in frequent repositioning of nucleosomes during evolution. Indeed, we show that nucleosome repositioning is both necessary and largely sufficient to explain the association between current nucleosome positions and biased substitution patterns. This finding highlights the importance of considering the direction of causality between genetic and epigenetic change.

## Author Summary

Genome sequences as well as epigenetic states, such as DNA methylation or nucleosome binding patterns, change during evolution. But what is the causal relationship between the two? We already know that nucleotide variation within and between species is distributed unevenly around nucleosome footprints, but does this mean that sequence evolution follows a biased course because the presence of nucleosomes affects mutation and DNA repair dynamics? Or is it, in fact, the other way around, i.e. changes happen at the DNA level and prompt shifts in nucleosome positioning? To investigate the direction of causality in genetic versus epigenetic evolution, we analyze substitutions patterns in eukaryotes as well as the archaeon *Haloferax volcanii* in the context of genome-wide nucleosome binding maps. We demonstrate that the relationship between nucleosome positions and between-species divergence patterns, strikingly similar in eukaryotes and archaea, can be explained in large parts by nucleosomes shifting positions in response to substitution, although both mutation and selection biases might still exist. Our results illustrate that it is important to consider the direction of causality between epigenetic and genetic change when analyzing patterns of sequence divergence and using sequence conservation to infer selection on epigenetic states.

## Introduction

Both *in vitro* and *in vivo*, nucleosomes are non-randomly positioned with regard to the underlying sequence, forming preferentially on stretches of DNA that – by virtue of their sequence composition – are more amenable to being wrapped around the histone core [1]–[3]. Consequently, changes at the sequence level during evolution can bring about changes in nucleosome positioning and occupancy [4], [5], providing a simple example of how genetic changes can locally alter epigenetic states.

Conversely, epigenetic states – here used in the broadest sense to include nucleosome positions, histone marks, DNA methylation state, etc. – can influence evolution at the sequence level. As applied to nucleosomes, three broad mechanisms can be distinguished. First, the presence of nucleosomes can affect the efficacy of DNA repair by altering the structural context in which lesions need to be detected and removed [6]. Second, rates of initial lesion formation can vary as a function of nucleosome occupancy. For example, a recent mutation accumulation experiment in yeast revealed a reduced incidence of C:G to T:A changes in nucleosome-bound regions [7], consistent with a model where DNA, when wound around a protein, is less likely to expose cytosine residues to conditions that promote spontaneous deamination. Third, since nucleosome positioning can mediate access to promoter elements or transcription factor binding sites [8], selection may eliminate mutations that alter nucleosome position in ways that disrupt proper access to these functionally important sites. In short, nucleosomes can affect evolution at the sequence level by modulating mutation and repair dynamics (thereby biasing the emergence of novel variants) and by exerting selective pressure on the underlying sequence (thereby altering fixation probabilities).

**Figure 1:**
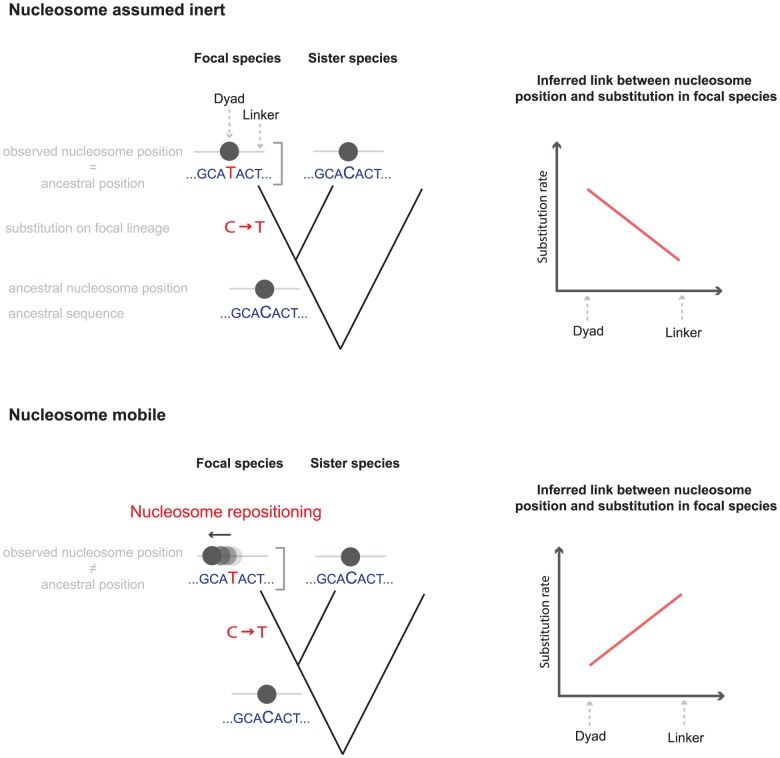
Interpreting the relationship between nucleosomes and substitutions. If nucleosome positioning does not change in response to a substitution (top panel), the assayed nucleosome position accurately represents the ancestral nucleosomal context in which the substitution occurred. In the case depicted here, we would correctly conclude that substitution rates are higher near the nucleosome dyad. If, on the other hand, the nucleosome had shifted following the substitution (bottom panel), whether directly in response to the substitution or prompted by other evolutionary changes in *cis* or *trans*, the ancestral relationship would no longer be reflected in the current data, leading to false conclusions.

Multiple recent studies have claimed support for either biased mutation or biased selection as the underlying cause behind (often strikingly) uneven divergence patterns around nucleosome in various eukaryotes including human, yeast, and *C. elegans* [7], [9]–[14]. For example, A:T to G:C substitutions were found to be more common closer to the nucleosome mid-point in humans, whereas C:G to T:A changes were enriched outside the nucleosome [9]. Interpretation of these trends has up to now proceeded from the assumption that experimentally determined nucleosome positions correspond closely to ancestral positions and that, as a result, current positions are informative about the chromatin context in which substitutions occurred. However, if this assumption is wrong and nucleosomes are repositioned following a change at the sequence level, conclusions about the relationship between nucleotide substitutions and nucleosome positions might change dramatically (Fig. 1). Might A:T to G:C substitutions, for example, be more common near the nucleosome dyad (the centre position of the binding footprint) simply because such a substitution tends, on average, to attract rather than repel nucleosomes?

Here, in an effort to disentangle cause and effect in the relationship between nucleosome binding and sequence evolution, we compare substitution patterns around nucleosome footprints in humans to substitution dynamics in the Haloferax clade, a group of halophilic archaea that includes the model haloarchaeon *Haloferax volcanii*, for which nucleosome organization was recently determined at high resolution [15]. Like their orthologs in eukaryotes, archaeal histones form multimeric complexes that preferentially assemble onto more bendable DNA templates [16], [17], with sequences bound *in vivo* exhibiting a higher average GC content in both eukaryotes and archaea [15], [18]. Further, nucleosome organization around *Hfx. volcanii* promoters is strongly reminiscent of nucleosome architecture in eukaryotes [15] suggesting that archaeal nucleosomes play similar roles in regulating access to DNA and controlling gene expression. Importantly, however, archaeal nucleosomes also differ in multiple respects from their eukaryotic counterparts: notably, although archaeal histones assemble into tetramers, homologous to the (H3–H4)_2_ tetramers seen in eukaryotes, they do not form octamers [19]. Consequently, archaeal nucleosomes are smaller and wrap less DNA, ∼85 nucleotides (nt) [17] compared to ∼147 nt in eukaryotes, with shorter linkers separating consecutive nucleosomes [15]. In addition, whereas eukaryotic histones sport N- and C-terminal tails, which can be acetylated, methylated or otherwise modified to generate distinct chromatin states, archaeal histones lack pronounced tails and there is currently no evidence for their post-translational modification [20].

Here, we demonstrate that – despite such differences in nucleosome structure, global nucleotide substitution profiles and general cellular physiology – nucleosome-associated substitution patterns along the *Hfx. volcanii* lineage are remarkably similar to those observed in humans (as well as in *Drosophila melanogaster*). In particular, strong similarities exist regarding which base-specific changes are more and which are less commonly observed when approaching the nucleosome dyad. We go on to show that these dyad-oriented trends break down in the respective sister lineages, an observation that is neither consistent with mutation nor purifying selection acting on nucleosomes that are positionally static over evolutionary time. Instead, we show that these patterns are consistent with widespread local repositioning of nucleosomes in response to substitutions. Our analysis provides a powerful caveat that the causal link between genetic and epigenetic change must be considered when assessing selection and mutation biases in the context of chromatin architecture.

## Results

### Similar substitution patterns in nucleosome footprints of eukaryotes and archaea

**Figure 2:**
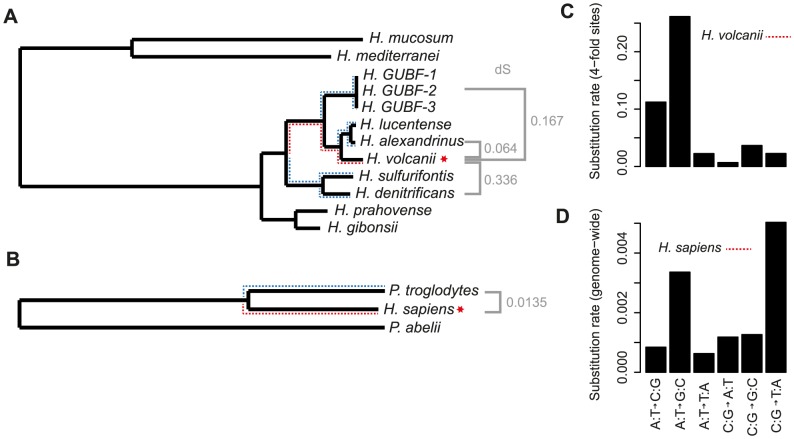
Phylogenetic relationships and global substitution profiles. Phylogenies for the two focal systems, Haloferax (A) and primates (B) are shown (see Materials and Methods for details about phylogenetic reconstruction). A red star marks those organisms for which experimental nucleosome data are available. Red and blue dotted lines indicate the lineages leading up to the two focal species and their sister lineages, respectively. These are the branches for which substitution rates were calculated (see Fig. 3 and Fig. 4). Global substitution profiles at 4-fold synonymous sites and total genomic sites are given for the *Hfx. volcanii* (C) and human (D) lineage, respectively.

In order to characterize nucleosome-associated substitution dynamics in *Hfx. volcanii*, we first identified orthologous protein-coding genes across 12 Haloferax genomes (see Materials and Methods). Following alignment, we reconstructed maximum likelihood phylogenies for each individual ortholog as well as for a concatenate of all orthologs. The concatenate-derived tree was taken to approximate the species tree (see Materials and Methods, Fig. 2a). As haloarchaea, including Haloferax, have a high propensity for horizontal gene transfer, as shown by both experimental studies [21] and phylogenomic analysis [22], we confined the reconstruction of substitution histories to 181 orthologs for which individual gene trees strictly reproduce the topology of the estimated species tree (see Materials and Methods). To avoid potential confounding effects from selection at the amino acid level, we further confined our analysis to changes that occurred at 4-fold synonymous sites between closely related species (Fig. 2a). We did not consider intergenic regions (which make up less than 15% of the Haloferax genome to begin with) because of the considerably greater uncertainty in orthology assignment. We also reconstructed substitutions along the human and chimp lineages using orangutan as the outgroup (see Materials and Methods, Fig. 2b). In this case, we considered substitutions in both coding and non-coding sequence, in part because we did not want to eliminate the contribution of promoter-associated nucleosomes, the principal candidates to be under selection for stable positioning (also see Materials and Methods).

Global substitution spectra in the two focal lineages (human and *Hfx. volcanii*, red branches in Fig. 2) differ markedly, with substitutions leading to *Hfx. volcanii* being heavily biased towards GC gains (Fig. 2c). The tendency for increased GC content at 4-fold synonymous sites is not restricted to the *Hfx. volcanii* lineage, but evident throughout the analyzed phylogeny (Fig. S1) and robust to outgroup identity (see Materials and Methods).

**Figure 3:**
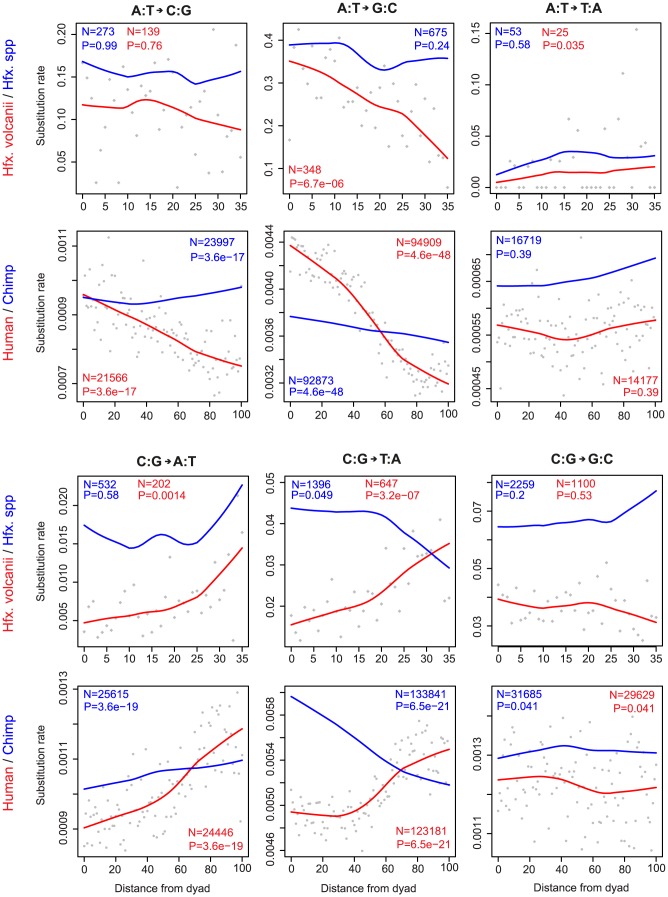
Substitution rates as a function of nucleosome topology. Nucleosome footprints in each genome (human, *Hfx. volcanii*) were lined up according to the inferred dyad and base-specific substitution rates along the focal (red) and sister (blue) lineages calculated at given distances (in nucleotides) from the dyad. Lines indicate LOWESS fits (smoother span f = 0.6), grey dots represent by-nucleotide-distance estimates for the focal lineages (omitted for clarity for the sister lineages). The number of substitutions (N) is given for each base change category along with P values for linear regression models, weighted by the number of eligible sites at each distance from the dyad. The maximum plotted distance from the dyad is chosen species-specifically to cover the typical nucleosome footprint plus neighbouring linker sequence in the different taxa.

Despite these radical differences in global substitution profiles, the specific effects of nucleosomes are remarkably similar. Considering base-specific substitution rates along the *Hfx. volcanii* lineage as a function of the distance to the nucleosome dyad, we recover trends that strongly resemble those observed on the human lineage [9] (Fig. 3). Substitution rates from weak (A or T) to strong (C or G) nucleotides are higher nearer the dyad, whereas the opposite is true for strong-to-weak changes, with little tendency in either direction shown by changes that preserve GC content.

### Nucleosome positions reflect sequence evolution

*A priori*, these shared trends are consistent with multiple models, including both shared mutational biases and a mixture of purifying and positive selection – an explanation advanced previously for human trends [9]. Alternatively, they could also reflect nucleosome repositioning in response to changes at the sequence level. This is because both archaeal and eukaryotic histone complexes share a preference for GC-rich sequence [15], [18] and might therefore show similar repositioning behaviour when the sequence context changes. In both primates and archaea, substitutions towards GC will, on average, attract rather than repel nucleosomes, leading to higher apparent rates of GC-enriching substitutions near the dyad.

We reasoned that it is possible to discriminate between these competing (but not necessarily mutually exclusive) hypotheses by comparing substitution trends along the focal branch (leading to the genome for which nucleosome positions have been experimentally determined) with substitution patterns along sister branches (blue branches in Fig. 2). To see why this is informative, consider the following scenario: let us assume that nucleosome positions are perfectly inert over the evolutionary short term so that, for instance, human and chimp nucleosomes would be in orthologous positions (a situation schematically represented in the left panel of Fig. 1). We could then analyze nucleotide changes along the chimp lineage as a function of human nucleosome positions projected onto orthologous chimp sequence. If mutational biases or selection were the sole causes behind the substitution trends in the human lineage, we would – with global mutation processes and selection regimes unlikely to differ substantially between human and chimp – expect to find substitution trends paralleling those observed in humans, with the same base-specific enrichment or depletion patterns around the dyad. If, on the other hand, nucleosome positions frequently shifted, possibly in response to substitutions, human and chimp dyads would often be found in non-orthologous positions, so that we might expect to observe quite different trends (if any) when we consider chimp substitutions as a function of human dyad positions. In fact, an evolutionary toy model (see Text S1) that explores which types of trends we would expect to see under different mutational and repositioning scenarios suggests that, in the absence of mutation or selection bias but with biased repositioning, we would frequently find strong trends in the focal species but no significant trend in the sister lineage. Less commonly, we might also observe trends in the sister lineage that go in the same or, more rarely yet, in the opposite direction as the focal trend. In contrast, under an assumption of no repositioning, we would always expect substitution trends in the sister lineage to parallel trends in the focal lineage, regardless of whether mutation rates were higher or lower in a nucleosomal context.

What do we observe empirically? When we project nucleosome positions onto aligned orthologous sequences in the respective sister lineages, we find that the strong signals observed in the focal lineages (Fig. 3, red lines) flatten out considerably, disappear altogether, or even invert (Fig. 3, blue lines). We also recover very similar patterns for Drosophila, when considering nucleosomes containing the histone variant H2AZ, mapped at high resolution in embryos of *Drosophila melanogster* [3] (Fig. S2).

There are two possible explanations for such divergent trends: first, mutation and/or selection processes are radically different in humans and chimps. In relation to mutation, there is no evidence that mutation processes differ to any noticeable degree in human versus chimp, *D. melanogaster* versus *Drosophila sechellia*, or *Haloferax volcanii* versus its sister lineages. Indeed, under a model of no repositioning, with only mutation bias as a potential culprit, we would have to evoke parallel changes in mutation bias along sister lineages from three independent and rather distinct clades in order to explain parallel trends in the respective focal versus sister lineages. This does not appear parsimonious. A closely analogous argument can be made to rule out selection as the major driver of dyad-related substitution trends: for functionally important nucleosomes, purifying selection should act in a similar manner in humans and chimps, so that we should see reduced rates of change in orthologous positions relative to the dyad. Although some divergent substitutions might be explained by positive selection in one of the two lineages, any contribution from nucleotides under positive selection will inevitably be dwarfed by nucleotides under a purifying regime. So the overwhelming prediction from a selection-based model would be to observe similar trends around nucleosomes in the focal and sister lineages. This we do not see.

In contrast, the empirical results are consistent with a repositioning-based model. In particular, the widespread absence of trends in the sister lineage mirrors expectations derived from our toy model where mutational/selective biases are not required to generate such trends. Further, the repositioning model offers a simple explanation for why weak-to-strong (GC-enriching) substitutions are more commonly found near the observed dyad, namely because, on average, they increased nucleosome formation potential relative to the ancestral sequence – in line with observed binding preferences towards GC-rich sequences [15], [18]. Conversely, strong-to-weak changes tend to reduce binding affinity so that the sites affected are now more likely than before to lie outside a current binding footprint.

### Repositioning is not random

We argued above that nucleotide changes and repositioning are causally linked. Need this necessarily be the case? *A priori*, our observations might also be consistent with a model where nucleosomes change positions in a non-sequence-dependent fashion, and subsequently affect the pattern of mutations, which, in turn, would lead to different trends in the sister lineage. However, this “wandering mutation bias” model does not stand up to closer examination. Let us assume, in line with this model, that nucleosomes did indeed move randomly (with regard to sequence context) and then affected the incidence of mutations. For us to observe *any* trend in the focal lineage (e.g. in human) under this scenario, nucleosomes would have to stay associated with the mutation they promoted (rather than randomly shift position again). If rates of shifting were high and sequence-independent, we would not observe any notable trend at equilibrium because the link between a nucleosome and the mutational skew it induces would be broken as often as generated. Conversely, if rates of shifting were low, the majority of nucleosomes would be in orthologous positions in chimp and human and exert their mutational bias on the same sequence, so that we would strongly expect to see similar trends caused by mutation bias in both chimp and human. In short, we do not think that random/non-sequence-specific nucleosome repositioning is consistent with the empirical evidence, i.e. clear-cut substitution trends in the focal but not the sister lineages. In contrast, our model of sequence-biased repositioning, which postulates a causal link between sequence change and nucleosome repositioning, does predict a) concordant trends in the focal lineages across clades, b) an absence of trends in the sister lineages, and c) neatly accounts for why we see GC-enriching changes enriched near the dyad (the nucleosome moved there) and GC-depleting changes enriched further away from the dyad (the nucleosome moved away).

It is important to highlight here that our results do not imply that there are no selection or mutation biases linked to nucleosome positioning. They do, however, strongly suggest that repositioning is necessary and also appears largely sufficient to explain the substitution trends we observe (see Discussion). As a result, future research on how mutational and selective biases affect substitution dynamics around nucleosome footprints should take into account the evolutionarily dynamic nature of nucleosome landscapes.

### A role for mutational bias in C:G to T:A patterns?

Our toy model suggested that, under a model solely driven by repositioning, we should mostly observe flat trends in the sister lineage. This is indeed the case for the majority of substitution types across taxa (Fig. 3). However, there is a conspicuous trend reversal for C:G to T:A changes, which prompted us to explore whether mutational bias might play a role in generating this particular trend. In humans, C:G to T:A changes derive primarily from deamination of methylated cytosine residues in a CpG context. If we exclude substitutions that happened in a CpG context, we retain a strong trend in humans but now find a flat trend in chimp (Fig. S3), suggesting that there was indeed a mutational bias, but one related to the higher frequency of CpGs inside of nucleosomes (in line with greater overall GC-richness), not one associated with the nucleosome *per se*. In Haloferax, disregarding changes at CpG dinucleotides does not alter the trend in the sister lineage (as we might expect given the absence of CpG methylation in this species), so this trend might be owing to strongly biased shifting or unknown mutational biases - we cannot currently distinguish between these two scenarios.

### A local repositioning model

The repositioning model implies that single nucleotide substitutions can bring about significant changes in nucleosome positioning. Although there is evidence for sequence-driven repositioning during evolution from comparative studies in yeast [4], [5], divergence levels between the yeast species analyzed are rather large (∼15% between *Saccharomyces cerevisiae* and its closest sequenced relative *Saccharomyces paradoxus*), meaning that, typically, several substitution have occurred within any one nucleosomal domain (spanning ∼147 nt plus flanking linker sequence). This makes it difficult to assess the impact of a single substitution on nucleosome positioning. In contrast, human-chimp divergence falls within a range where many nucleosomal domains have only experienced a single substitution since the two lineages split.

To gain further insights into the effects of single substitution on nucleosome positioning (and in the absence of experimental data on nucleosome positions in chimp), we therefore assessed predicted nucleosome formation potential of reconstructed ancestral sequence and compared it to predictions for the derived human sequence.

**Figure 4:**
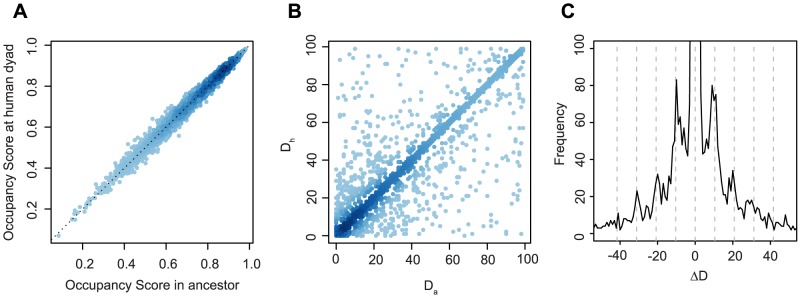
Comparing predicted ancestral and predicted extant nucleosome occupancy. (A) The relationship between nucleosome occupancy scores as predicted for nucleotides positioned at nucleosome dyads in humans and the corresponding nucleotides in the ancestor of humans and chimps (see Materials and Methods for details on the prediction algorithm). (B) The distance between each human dyad and the nucleotide with the highest occupancy score within a ±100 nt window around that dyad as calculated for human sequence (D_h_) and ancestral sequence (D_a_) assuming human dyad positions. Only dyads where a single substitution had occurred within the ±100 nt window along the human lineage were considered. (C) Distribution of differences (ΔD) between D_a_ and D_h_ as defined in the text.

First, we confirmed that weak-to-strong changes do, in fact, tend to increase nucleosome occupancy scores whereas the reverse is true for strong-to-weak changes (Fig. S4, see Materials and Methods). The magnitude of that change, however, is small compared to the global spread of occupancy scores (Fig. 4A). In other words, a single substitution rarely turns a favourable sequence into an unfavourable one, perhaps suggesting that radical eviction is relatively rare and subtle repositioning to neighbouring translational positions more common. To explore how positioning landscapes might change on a more local level, we considered the distance between experimentally defined dyads and the nucleotide with the highest occupancy score within 100 nt either side of each dyad in human (D_h_) and in the ancestor (D_a_). The difference between these distances (ΔD) serves as measure of local shifts in nucleosome formation potential from ancestral to human sequence. This analysis, using the human dyad as a convenient reference point, aims to explore whether positioning might change locally using occupancy as a coarse proxy for where nucleosomes are most likely to form.

**Figure 5:**
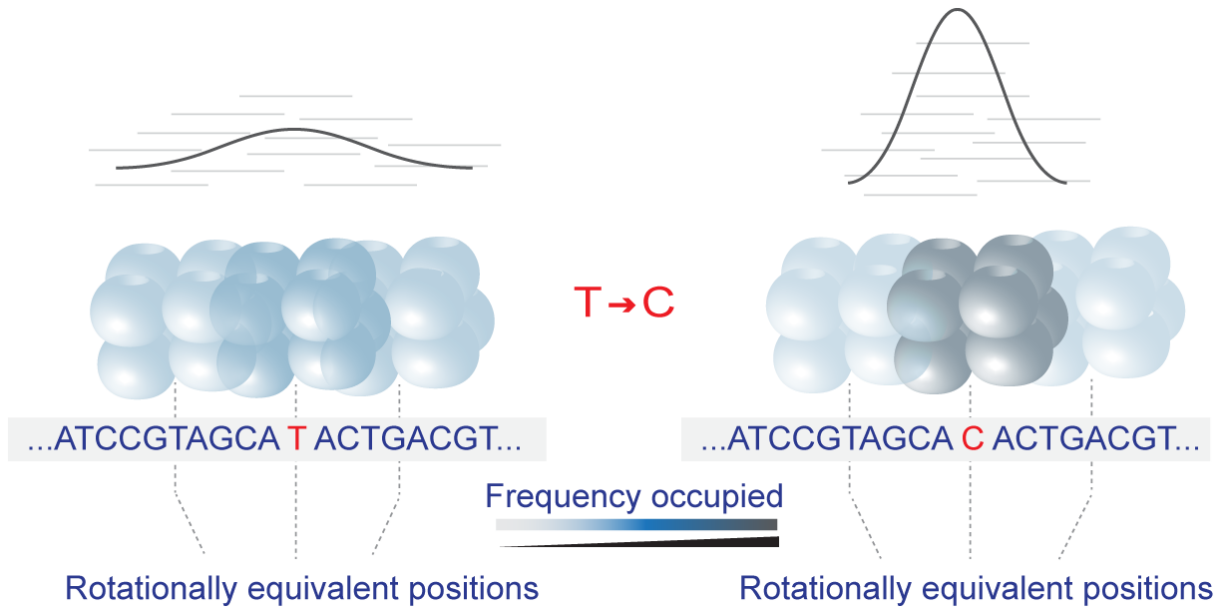
Reweighting of local positioning landscapes. In a population of cells (in space or time), nucleosomes can statistically occupy partially overlapping, rotationally equivalent positions. Some rotational positions can be more frequently occupied than others. This may be related, in part, to differences in the affinity of the underlying sequence. If such differences are subtle, assayed positions will be found spread relatively evenly across rotationally equivalent positions. A substitution can alter the local distribution of affinities, strengthening (as shown here) or weakening the nucleosome formation potential of the underlying sequence. As a consequence, positioning across the population might be skewed towards a specific translational position, making it more likely that that position is identified as being occupied by a nucleosome.

Focusing on regions where a single substitution occurred within a ±100 nt window around the dyad, it emerges that local changes regarding which local sequence context is most amenable to nucleosome formation are relatively common, ΔD exceeding 10 nt in 5.1% of the cases (Fig. 4B). Notably, where the site of highest predicted occupancy differs between ancestor and human, the shift size appears non-randomly enriched for multiples of ∼10 nt either side of the dyad (Fig. 4C). This tentatively suggests that substitutions strengthen alternative positions in the vicinity of the ancestral dyad that are rotationally equivalent. This would be in line with experimental evidence that translational positioning is rather flexible locally, but that rotational positions are typically maintained, leading – when considering positions across a population of cells - to a statistical array of overlapping centre positions spaced by ∼10 nt [23]. Attempts to call a defined dyad from such a population-based signature (to the exclusion of overlapping peaks) will select, on average, dyad positions with the highest proportional occupancy in the population. Note that, in the absence of a well-defined random expectation, it is difficult to formally test whether this apparent enrichment is significantly different from what we would expect to see by chance. However, based on the absence of radical changes in occupancy caused by individual substitutions (Fig. 4A), our current favoured model (Fig. 5) is one where substitutions reweight local occupancy landscapes, by strengthen or weakening the affinity for certain rotationally equivalent positions, making it more or less likely for a dyad to be called at that site. On average, changes towards increased GC content are more likely to generate attractor states that promote nucleosome formation, increasing the chance that the dyad position is called at or near where the change occurred, whereas the reverse is true for changes towards AT, which are more likely to disfavour nucleosome formation.

## Discussion

Our analysis revealed that substitution patterns in and around nucleosome footprints are remarkably similar along the *Hfx. volcanii* and human branches, with substitution rates consistently reduced for some (e.g. C:G to T:A) but elevated for other (e.g. A:T to G:C) base changes when approaching the dyad. These shared biases are observed despite radical differences in global substitution dynamics and structural differences between the histone complexes involved. Exploring whether common factors generate these signatures in both clades, we discovered that substitution trends in the respective sister lineages do not show the same behavior, an observation that is inconsistent with mutational biases or purifying selection acting on nucleosomes that are positionally inert through evolution. On the contrary, our analysis demonstrates that many nucleosomes must have repositioned from their ancestral locations. Further, we argue that biased repositioning is largely sufficient to explain the association between nucleosome topology and nucleotide substitution rates, whereby nucleosomes shift (likely locally) from their ancestral position in response to a change in the underlying sequence, and do so in line with known histone binding preferences.

The results presented here also highlight that defining a unique dyad, rather than considering a partly overlapping, probabilistic ensemble of nucleosome footprints, might lead to misleading conclusions because sequence changes – while not affecting the local ensemble of positions *per se* – can affect which translational positions are occupied more frequently and hence affect which of these positions is picked when calling the dyad.

Our findings do not imply that mutations are not modulated by nucleosome occupancy or that selection does not act to maintain at least a subset of nucleosomes in functionally relevant positions. In fact, recent results from mutation accumulation lines in yeast [7] strongly support the notion that there are systematic differences in mutation rates for sequences bound by nucleosomes versus linker DNA. A role for mutation and/or selection also appears supported by the biased incidence of single nucleotide polymorphisms around nucleosomes in human and yeast [12], [14], [24], [25], although the possible effect of different ancestral nucleosome positions was not considered in these studies.

What our findings do suggest, however, is that mutation/selection biases are not sufficient to explain the observed association between nucleosome positions and nucleotide substitution patterns between species and must operate, if they do, on a background of nucleosome repositioning. As a result, our analysis provides an important general caveat to interpreting substitution dynamics in relation to observable epigenetic states because these states might have been different at the time when the substitutions occurred. Future analyses, especially when concerned with detecting signatures of purifying and, above all, positive selection, should take nucleosome mobility into account and ideally model explicitly how mutation biases, selection, and re-positioning interact to determine the co-evolution of nucleosome positions and the underlying sequence. Such studies should focus on closely related sister taxa as – with increasing evolutionary distance – the causal relationship between genetic and nucleosome positional change will become increasingly harder to decipher. This is principally because multiple nucleotide changes, which may affect binding in a hard-to-predict combinatorial fashion, need to be considered concurrently. Indeed, comparing *S. cerevisiae* and *S. paradoxus*, which are substantially further diverged than the primate, Drosophila and Haloferax sister pairs analyzed above, we do not recover analogous trends at 4-fold synonymous sites, despite the availability of high-resolution nucleosome datasets (Fig. S5). In addition, studies of the type conducted by Chen and colleagues [7], where ancestral footprints can be assayed directly and changes followed forward in time will be invaluable to learn more about mutation biases *in vivo* and understand, for example, whether mutational biases largely reinforce current positioning (as might be the case for reduced C:G to T:A rates inside of nucleosomes [7], where changes towards nucleosome-disfavouring, AT-rich sequence would be concentrated in already disfavouring sequence) or indirectly favour positional stability over the longer term by promoting compensatory mutation in the regions that assume a new mutation regime when the nucleosome is repositioned.

## Materials and Methods

### Ortholog identification and alignment

We obtained Haloferax genome sequences and coding sequence annotations from multiple sources listed in Table S1. Annotated coding sequences that i) did not contain ambiguous nucleotides, ii) were a multiple of three nucleotides long and iii) did not contain internal stop codons were translated into protein. These *in silico* proteomes were blasted against each other, reciprocal best hits retained (Protein-Protein BLAST 2.2.24+, minimum E-value: 0.001), and an initial list of orthologs defined based on consistent reciprocal hits across all 12 Haloferax genomes. These candidate orthologs were aligned at the protein level using Muscle (version 3.8.31) [15], [26]. Orthologs with >70% sequence identity and <5% length difference across all pairwise comparisons were retained for further analysis and back-translated to nucleotides.

### Phylogenetic reconstruction and analysis

Aligned coding sequences were concatenated and submitted to PhyML (version 3.0) [20], [27]. The topology of the resulting tree (Fig. 2) is consistent with previously published trees that contained a subset of the genomes analyzed here [22], [28]. Note that the GUBF strains are so closely related that we consider this part of the tree as an unresolved polytomy. Any of the three GUBF strain can be dropped from the analysis without affecting the results reported here. In analogous fashion, we also built gene trees for individual orthologs and our analysis of substitution rates conservatively only includes genes whose topology matches that of the species tree. Substitution patterns along the phylogeny were then inferred using PAML [29].

We tested whether outgroup identity might unduly affect substitution rate estimates by dropping *Haloferax mediterranei* and *Haloferax mucosum* from the analysis and, in a second, independent test by confining analysis to the triplet *Hfx. volcanii/Haloferax alexandrinus/Haloferax lucentense*, inferring changes along the *Hfx. alexandrinus* and *Hfx. lucentense* branches by parsimony. In both cases, global GC-biased substitution spectra remain qualitatively unchanged (Fig. S6).

### Eukaryotic data

To replicate the analysis of Prendergast and Semple [9] we downloaded genome-wide human-chimp and human-orangutan alignments from UCSC (Table S1), linked them using the human coordinates, and called changes along the human and chimp lineages by parsimony. We ignored sites where nucleotide identity in the outgroup did not agree with either the chimp or human nucleotide. The human-chimp ancestral sequence, reconstructed from 4-way (human-chimp-orangutan-macaque) alignments, was downloaded from the 1000 Genomes Project (Table S1).

Drosophila multiple alignments were downloaded from UCSC (Table S1) and processed in a fashion analogous to the procedure for primates. *Drosophila erecta* and *Drosophila yakuba* served as a joint outgroup (see Fig. S2). Analysis was confined to four-fold synonymous sites, defined according to coding sequence annotations from FlyBase (Table S1).

Yeast sequences and ortholog assignments were obtained from the Broad Institute (Table S1) and alignments constructed as described for Haloferax, with the exception that no cut-offs were imposed regarding similarity or protein length differences.

### Nucleosome data and prediction of ancestral nucleosome positions

Dyad calls were obtained from original publications (Table S1). To calculate nucleosome occupancy scores in human, we extracted each dyad position (or the homologous position in the ancestor, respectively), along with 5000 nucleotides up- and downstream, and ran the nucleosome occupancy prediction algorithm from the Segal lab (http://genie.weizmann.ac.il/software/nucleo_exe.html) (version 3) on each such sequence fragment. We ignored fragments that contain ambiguous nucleotides.

Where necessary, coordinates were converted to hg19 using the LiftOver tool at UCSC (http://genome.ucsc.edu/cgi-bin/hgLiftOver).

### Analysis of 4-fold synonymous sites in primates

As the subset of human nucleosomes used by Prendergast and Semple [9] and originally called by Reynolds et al. [30] predominantly contains nucleosomes in non-coding regions, we decided not to confine analysis to 4-fold synonymous sites as we did for Haloferax. However, we note that the same patterns are also evident, despite reduced statistical power, when analysis is restricted to 4-fold synonymous sites (Fig. S7), with AT-enriching substitutions along the human lineage more common further away from the dyad and GC-enriching changes less common (with no significant trend for A:T to C:G where we have few observations and lack statistical power). Changes that maintain GC content (A:T to T:A and G:C to C:G) show no significant trends as observed when including all sites.

## Supporting Information

Figure S1 **Branch-specific substitution spectra.** Numbered substitution profiles correspond to branch labels on the Haloferax phylogeny.
(EPS)

Figure S2 **Drosophila phylogeny, substitution profiles and the relationship between nucleosome dyads and substitution rates.** Focal (*D. melanogaster*) and sister (*D. sechellia*) lineages are colour-coded analogous to Fig. 3. Global substitution profiles at 4-fold synonymous sites are shown for both focal and sister lineage. The *D. melanogaster* lineage exhibits increased rates of C:G to T:A substitutions, as previously observed [31].
(EPS)

Figure S3 **Substitution profiles for C:G to T:A changes with CpG dinucleotides removed.** C:G to T:A substitution along the human (red) and chimp (blue) lineages where the ancestral C was not present in a CpG context. Weighted linear regression: P(chimp) = 0.003; P(human) = 5.38*10^−32^.
(EPS)

Figure S4 **Weak-to-strong and strong-to-weak substitutions alter predicted occupancy scores in the expected directions.** The difference (Δ) between predicted human and predicted ancestral occupancy scores is shown as a function of the distance of a given substitution from the human dyad. Weak-to-strong substitutions located close to an extant dyad typically increased occupancy scores, whereas those further away did not have that effect.
(EPS)

Figure S5 **Saccharomyces phylogeny, substitution profiles and the relationship between nucleosome dyads and substitution rates.** Focal (*S. cerevisiae*) and sister (*S. paradoxus*) lineages are colour-coded analogous to Fig. 3. Nucleosome-associated trends are shown for 4-fold synonymous sites and all coding nucleotides according to the dyad catalogues of Weiner et al (2010) [32] and Brogaard et al. (2012) [33]. Substitution trends at 4-fold synonymous sites largely do not follow the pattern established in Drosophila, primates, and Haloferax. Interestingly, considering all coding nucleotides, the two datasets disagree in their placement of nucleosome dyads relative to substitutions. It is worth highlighting that, for several base change categories (e.g. A:T to T:A), substitution rates are notably higher a multiple of three nucleotides from the dyad in the coding/Brogaard data, suggesting that nucleosomes in yeast might be non-randomly positioned relative to the reading frame of protein-coding genes. Vertical dashed lines are spaced at intervals of n = 0, 3, … 99 nucleotides from the dyad.
(EPS)

Figure S6 **The effect of outgroup identity on global substitution profiles.** (A) Global substitution profiles at 4-fold synonymous site along the *Hfx. volcanii* lineage when orthologs were identified and substitutions reconstructed omitting *Hfx. mediterranei* and *Hfx. mucosum*. (B) Global substitution profiles at 4-fold synonymous site along the *Hfx. alexandrinus* and *Hfx. lucentense* lineages inferred by parsimony with *Hfx. volcanii* as the outgroup.
(EPS)

Figure S7 **Substitution rates at 4-fold synonymous sites in primates as a function of nucleosome topology.** Nucleosome footprints in the human genome were lined up according to the inferred dyad and base-specific substitution rates at 4-fold synonymous sites along the human (red) and chimp (blue) lineages calculated at given distances (in nucleotides) from the dyad. Lines indicate LOWESS fits (smoother span f = 0.6), grey dots represent by-nucleotide-distance estimates for the focal lineages (omitted for clarity for the sister lineages). P values are for linear regression models, weighted by the number of eligible sites at each distance from the dyad. As the set of nucleosomes used in the main analyis (SRest80) poorly overlaps coding sequences, we used a larger, less stringent set (SRest50) called by Reynolds et al [30] for the very same experimental data.
(EPS)

Table S1 **Data sources.**
(DOCX)

Table S2 **Simulation results for the evolutionary toy model.** See Text S1 for a detailed description.
(TXT)

Text S1 **An evolutionary toy model to explore nucleosome repositioning.**
(PDF)
