# Supplementary material for: Conserved Substitution Patterns around Nucleosome Footprints in Eukaryotes and Archaea Derive from Frequent Nucleosome Repositioning through Evolution
Source: PLoS Comput Biol. 2013 Nov 21;9(11):e1003373. doi: 10.1371/journal.pcbi.1003373 (PMC3836710; doi:10.1371/journal.pcbi.1003373)
Supplement: Table S1 — Data sources. (DOCX) [file pcbi.1003373.s008.docx]

| **Genomes, alignments & nucleosome data** | **Source** | **Web link (if applicable)** |
| --- | --- | --- |
| *H. volcanii* DS2  *H. mediterranei* ATCC 33500 | NCBI | ftp.ncbi.nih.gov/genomes/Bacteria/Haloferax_volcanii_DS2_uid46845/ |
| *H. mucosum*  *H. sulfurifontis*  *H. denitrificans* | Edwards lab | http://edwards.sdsu.edu/halophiles/ |
| *H. alexandrinus*  *H. lucentense*  *H. prahovense*  *H. gibonsii*  *H.* GUBF-1  *H.* GUBF-2  *H.* GUBF-3 | Facciotti lab | * |
| Human-chimp alignment | UCSC | http://hgdownload.cse.ucsc.edu/goldenPath/hg19/vsPanTro3/axtNet/ |
| Human-orangutan alignment | UCSC | http://hgdownload.cse.ucsc.edu/goldenPath/hg19/vsPonAbe2/axtNet/ |
| Human-chimp ancestral sequence (reconstructed from 4-way human-chimp-orang-macaque alignment) | 1000 Genomes Project | ftp://ftp.1000genomes.ebi.ac.uk/vol1/ftp/pilot_data/technical/reference/ancestral_alignments/ |
| Drosophila multiple species alignment | UCSC | http://hgdownload.cse.ucsc.edu/goldenPath/dm3/multiz15way/ |
| Drosophila coding sequence annotations | FlyBase | dmel-all-CDS-r5.48.fasta |
| Saccharomyces sequences | Broad Institute | http://www.broadinstitute.org/annotation/fungi/comp_yeasts/downloads.html |
| Saccharomyces ortholog assignments | Broad Institute | http://www.broadinstitute.org/regev/orthogroups/ |
| *S. cerevisiae* functional annotations | SGD | http://downloads.yeastgenome.org/sequence/S288C_reference/genome_releases/ |
| Dyad calls for human CD4+ T-cells | Noble lab | http://noble.gs.washington.edu/papers/reynolds2010learning.html |
| Dyad calls for *H. volcanii* | Nislow lab | http://chemogenomics.med.utoronto.ca/supplemental/chromatin/files/Archaeal_browser_track_2N.bed |
| Dyad calls for *D. melanogaster* | Penn State Genome Cartography project | http://atlas.bx.psu.edu/data/dmel/ |
| Dyad calls for *S. cerevisiae* | Friedman lab | http://compbio.cs.huji.ac.il/NucPosition/TemplateFiltering/Nucleosome_calls.html  (BY 10uL normal digestion) |
|  | Brogaard et al (2012) [33] | Supplementary Table 2 |

*These Whole Genome Shotgun projects have been deposited at DDBJ/EMBL/GenBank under the accessions

AOLD00000000 *Haloferax sp.* ATCC BAA-646 [GUBF-3]

AOLE00000000 *Haloferax sp.* ATCC BAA-645 [GUBF-2]

AOLF00000000 *Haloferax sp.* ATCC BAA-644 [GUBF-1]

AOLG00000000 (*Haloferax prahovense* DSM 18310)

AOLH00000000 (*Haloferax lucentense* DSM 14919)

AOLJ00000000 (*Haloferax gibbonsii* ATCC 33959)

AOLL00000000 (*Haloferax alexandrinus* JCM 10717)

The versions described in this paper are the first versions, AOLD01000000, AOLE01000000, AOLF01000000, AOLG01000000, AOLH01000000, AOLJ01000000, and AOLL01000000, respectively.
