# Supplementary material for: Conserved Substitution Patterns around Nucleosome Footprints in Eukaryotes and Archaea Derive from Frequent Nucleosome Repositioning through Evolution
Source: PLoS Comput Biol. 2013 Nov 21;9(11):e1003373. doi: 10.1371/journal.pcbi.1003373 (PMC3836710; doi:10.1371/journal.pcbi.1003373)
Supplement: Text S1 — An evolutionary toy model to explore nucleosome repositioning. (PDF) [file pcbi.1003373.s010.pdf]

An evolutionary toy model to explore nucleosome repositioning

We decided to investigate what types of substitution patterns (particularly with regard to the sister lineage) we should expect under different evolutionary scenarios (with/without shifting and/or mutational bias). To do so, we built a simple evolutionary toy model. We start with a simple "chromatin unit" that consists of two neighbouring segments, one in a "nucleosome state", the other in a "linker state" (Figure S8).

We then let this chromatin unit mutate and nucleosomes reposition using 4 parameters: $P(mut)_{nuc}$, the probability that a mutation occurs in the nucleosome state, $P(mut)_{link}$, the probability that a mutation occurs in the linker state, $P(shift)_{nuc}$, the probability that a nucleosome will "shift" out of the nucleosome state into a linker state when a mutation occurs in the nucleosome segment, and $P(shift)_{link}$, the probability that a nucleosome shifts into the linker segment if a mutation occurs in the linker. Shifting here simply implies a change of states that is reciprocal: if a nucleosome shifts, the nucleosome state becomes the linker state and the neighbouring linker state becomes a nucleosome state.

We simulate 100,000 such units, mutate each independently (maximum one mutation per unit) and track a) if a mutation occurred, b) in which ancestral context it occurred (in the linker or nucleosome state), and c) in which context it is now observed (i.e. a mutation might occur in the linker segment, but that segment might now be in a nucleosomal state if the nucleosome shifted). For each unit that we evolve, we have a matching partner (the "chimp unit"), which we evolve under the same parameters. Critically, we can now evaluate where mutations occurred in chimp relative to the observed human state (which, if the nucleosome moved, can be different from the chimp and/or ancestral state), exactly like we do empirically for the substitution data.

An example is illustrated in Figure S8 for a model where the probability of mutation is the same in nucleosome and linker state ($P(mut)_{nuc}= P(mut)_{link}$), but where a shift only occurs if the mutation falls in a nucleosomal region (much as we propose might frequently be the case for C to T substitutions). In our example, we consider two chromatin units in parallel (to illustrate equal mutation rates in nucleosome and linker). The first unit (unit A) mutates in the nucleosome state in humans, causing a shift, whereas the orthologous unit in chimp mutates in the linker region causing no shift. The opposite happens for unit B. We can now ask, across units A and B, how many mutations we observe in the current linker and nucleosome state in humans (top left panel). More importantly, we can ask where mutations fall in chimp relative to the current human nucleosome state (grey arrows). In this example, we observe both chimp mutations in a nucleosome state, although one of them actually occurred in the linker state. Evidently, we might also observe cases where mutations fall in the same segment in both human and chimp (grey shaded box) so that we would end up observing similar trends in both species. On average (dashed box) we would observe a strong trend in humans but no trend in chimps.

Figure S8

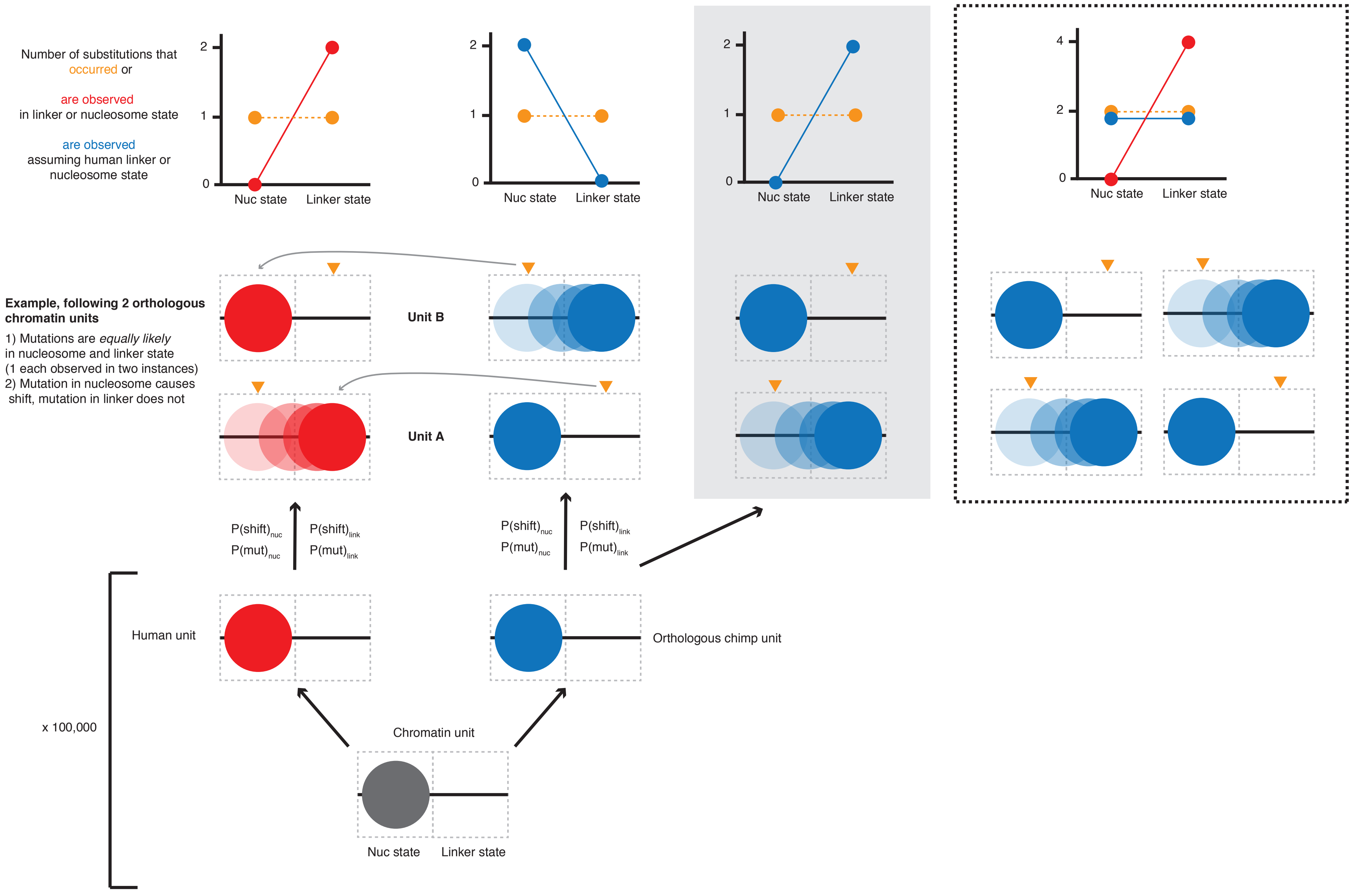

Number of substitutions that
occurred or
are observed
in linker or nucleosome state
are observed
assuming human linker or
nucleosome state
2
1
0
Nuc state
Linker state
4
Example, following 2 orthologous chromatin units
1) Mutations are *equally likely* in nucleosome and linker state (1 each observed in two instances)
2) Mutation in nucleosome causes shift, mutation in linker does not
Unit B
Unit A
P(shift)nuc
P(mut)nuc
P(shift)link
P(mut)link
Human unit
Orthologous chimp unit
x 100,000
Chromatin unit

Using different combinations of values for the four parameters, we then consider the following: If $P(mut)_{nuc}= P(mut)_{link}$, i.e. there is no nucleosome-associated mutation bias, can we replicate the array of trends we observe empirically simply by having differential rates of nucleosome shifting following mutation in the nucleosome and linker state?

The model reveals – as fore-shadowed by the example given in Figure S8 – that different rates of mutation-induced shifting is sufficient to generate patterns with strong trends in human, but flat trends in chimp, where the direction and steepness of the human trend depends on the absolute and relative shifting frequencies. A representative sample of parameter combinations is given in Figure S9.

In addition, under some parameter combinations we can also obtain non-flat trends in chimps *even in the absence of mutation bias*, both in the same direction as the human trend, but also, more strikingly, in the opposite direction (see Figure S10).
For most parameter combinations (under $P(mut)_{nuc}= P(mut)_{link}$) we see flat trends in chimps, which is what we observe empirically. These results highlight that under a model without mutation bias and solely based on differential shifting we largely expect to observe trends in human but no trends in chimp, but might more rarely observe trends in chimp too. A full set of simulated substitution rates can be found in Table S2.

Figure S9

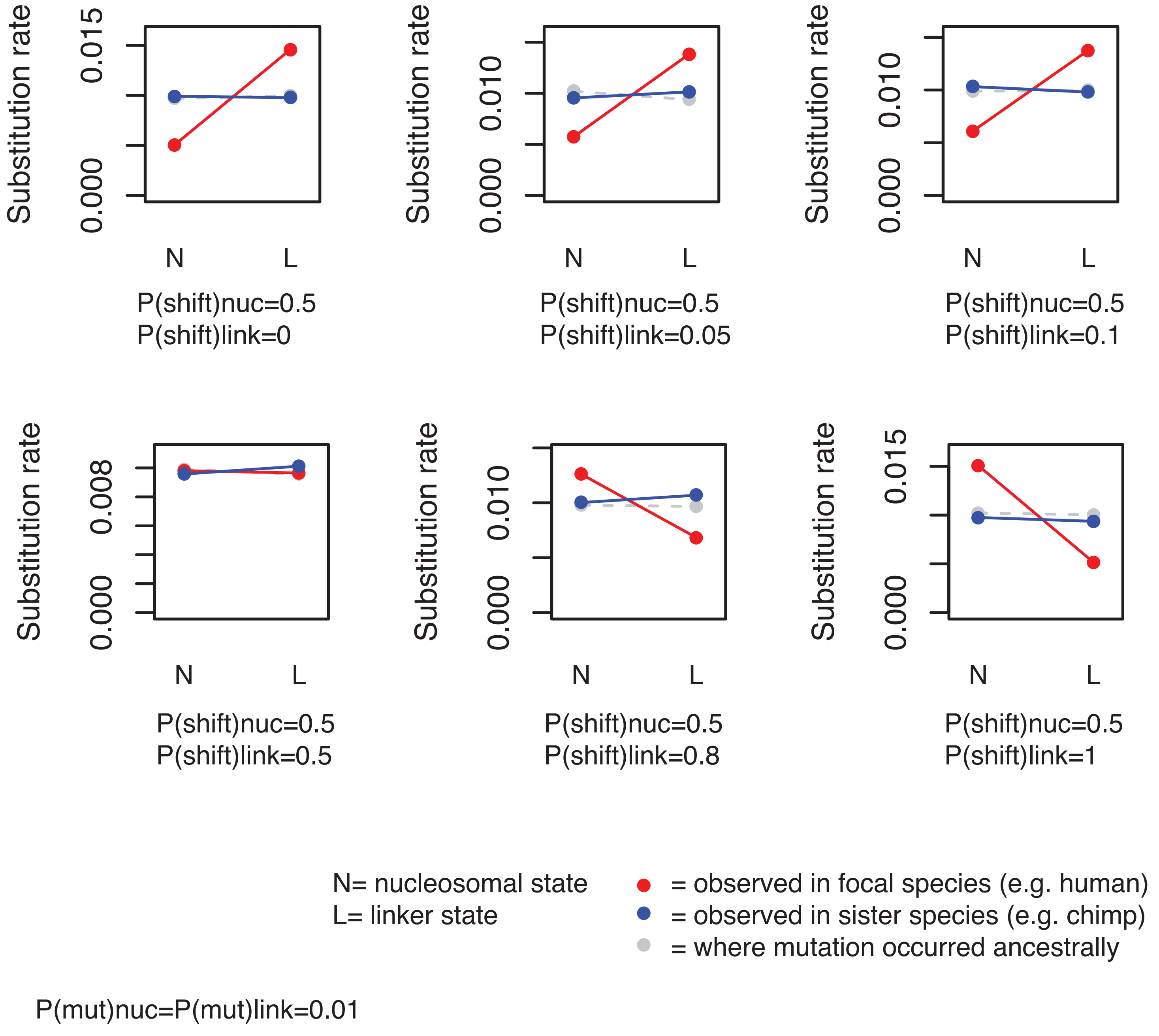
Substitution rate
0.000
0.015
N
L
P(shift)nuc=0.5
P(shift)link=0
Substitution rate
0.000
0.010
N
L
P(shift)nuc=0.5
P(shift)link=0.05
Substitution rate
0.000
0.010
N
L
P(shift)nuc=0.5
P(shift)link=0.1
Substitution rate
0.000
0.008
N
L
P(shift)nuc=0.5
P(shift)link=0.5
Substitution rate
0.000
0.010
N
L
P(shift)nuc=0.5
P(shift)link=0.8
Substitution rate
0.000
0.015
N
L
P(shift)nuc=0.5
P(shift)link=1
N= nucleosomal state
L= linker state
= observed in focal species (e.g. human)
= observed in sister species (e.g. chimp)
= where mutation occurred ancestrally
P(mut)nuc=P(mut)link=0.01

Figure S10

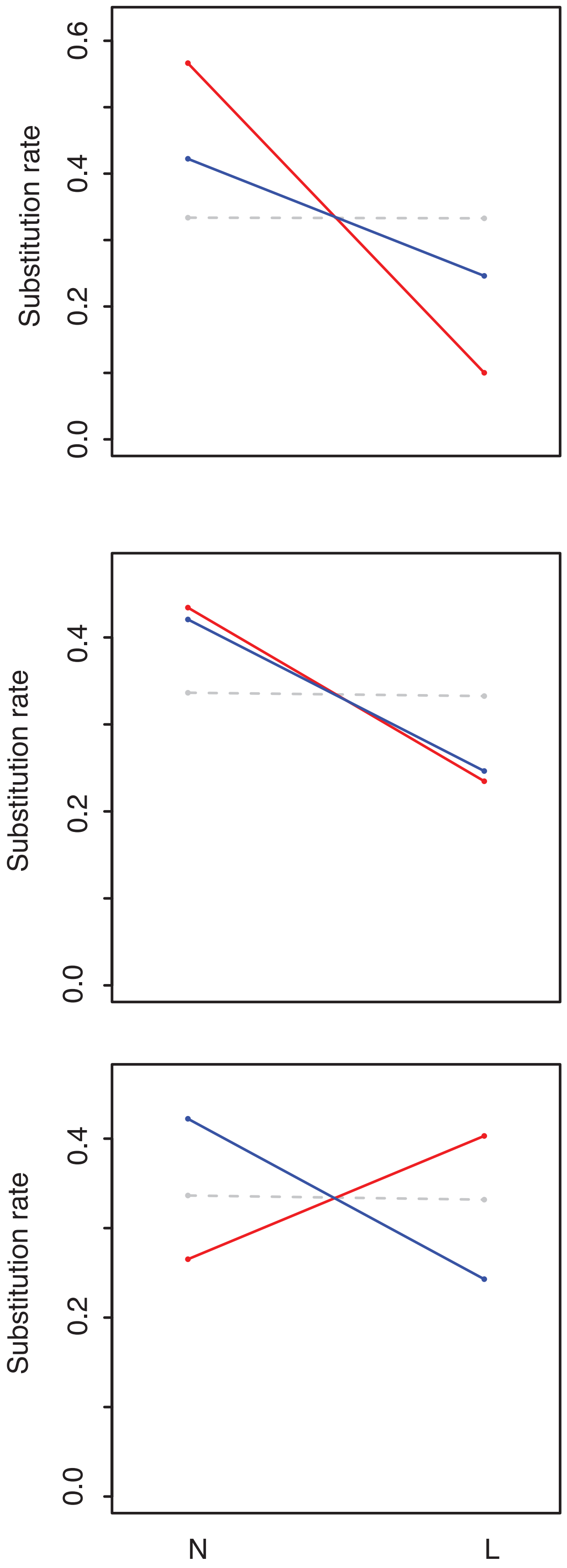
Substitution rate
0.0
0.2
0.4
0.6
Substitution rate
0.0
0.2
0.4
Substitution rate
0.0
0.2
0.4
N
L
